# Induction Chemoimmunotherapy Versus Induction Chemotherapy Followed by Concurrent Chemoradiotherapy for Locally Advanced Esophageal Squamous Cell Carcinoma: A Propensity Score-Matched Study

**DOI:** 10.3390/jcm15208121

**Published:** 2026-10-19

**Authors:** Jiayue Liu, Wei Deng, Xin Dong, Yuting Zhao, Leilei Jiang, Liuhua Long, Yue Teng, Dan Yang, Jiayi Yu, Xiao Chang, Huiming Yu, Anhui Shi, Weihu Wang, Rong Yu

**Affiliations:** Key Laboratory of Carcinogenesis and Translational Research (Ministry of Education), Department of Radiation Oncology, Peking University Cancer Hospital and Institute, Beijing 100142, China

**Keywords:** esophageal squamous cell carcinoma, induction chemoimmunotherapy, induction chemotherapy, concurrent chemoradiotherapy, immunotherapy, propensity score matching

## Abstract

**Background/Objectives:** Induction chemotherapy offers limited survival benefit before definitive concurrent chemoradiotherapy in locally advanced esophageal squamous cell carcinoma. We compared induction chemoimmunotherapy followed by concurrent chemoradiotherapy (IOC-CCRT) with induction chemotherapy followed by concurrent chemoradiotherapy (IC-CCRT). **Methods:** This retrospective study included patients treated during 2017–2024. Propensity score matching was performed at a 2:1 ratio. Overall survival, progression-free survival, failure patterns, and treatment-related adverse events were evaluated. **Results:** Among 333 patients, 228 received IOC-CCRT and 105 received IC-CCRT. In the full cohort, IOC-CCRT was associated with better overall survival (*p* = 0.009) and progression-free survival (*p* = 0.005). After matching, 220 patients were included (141 IOC-CCRT and 79 IC-CCRT); IOC-CCRT remained associated with better overall survival (*p* = 0.0027) and progression-free survival (*p* = 0.0012). The 36-month overall survival rates were 59.0% versus 41.6%, and progression-free survival rates were 49.7% versus 28.0%. Overall failure occurred in 27.7% versus 39.2% of patients (*p* = 0.077). In multivariable analyses, the associations with progression-free survival (hazard ratio, 0.75; 95% confidence interval, 0.55–1.03; *p* = 0.075) and overall survival (hazard ratio, 0.82; 95% confidence interval, 0.58–1.14; *p* = 0.234) were not statistically significant. Grade 3 or higher neutropenia was more frequent with IOC-CCRT (31.9% versus 16.5%; *p* = 0.013); no grade ≥ 3 radiation pneumonitis occurred. **Conclusions:** IOC-CCRT was associated with improved unadjusted survival outcomes and increased selected severe toxicities compared with IC-CCRT. Prospective randomized trials are warranted.

## 1. Introduction

Definitive concurrent chemoradiotherapy (CCRT) is the standard of care for patients with unresectable locally advanced esophageal squamous cell carcinoma (ESCC). Esophageal squamous cell carcinoma remains the predominant histologic subtype worldwide, accounting for the majority of esophageal cancer cases [1,2,3,4]. Despite established combined-modality therapy, durable control remains difficult, with both locoregional and distant failures contributing to poor long-term outcomes [5,6,7,8,9,10].

Induction chemotherapy before CCRT has been used to decrease tumor burden before definitive local therapy. By reducing primary tumor volume and controlling occult micrometastatic disease at an early stage, induction chemotherapy may improve treatment tolerance, facilitate subsequent radiotherapy planning, and enhance locoregional control, particularly in patients with bulky or unresectable locally advanced disease [11,12]. On the basis of these potential advantages, induction chemotherapy has been increasingly incorporated into multimodality treatment strategies for patients with locally advanced ESCC [12,13]. Despite these theoretical benefits, its clinical value has remained controversial. A randomized phase II trial in ESCC found no survival advantage for induction chemotherapy followed by definitive chemoradiotherapy over CCRT alone in an unselected population [14]. Additional clinical studies and meta-analyses have also reported inconsistent findings [15,16,17]. Collectively, these data suggest that the effectiveness of induction treatment may depend not only on its timing but also on the therapeutic agents incorporated into the induction regimen.

Immune checkpoint inhibitors have changed the treatment landscape of ESCC. In advanced disease, several phase 3 trials have shown that programmed cell death protein 1 (PD-1) blockade combined with chemotherapy improves survival compared with chemotherapy alone [18,19,20,21,22,23]. In resectable disease, neoadjuvant chemoimmunotherapy has also shown substantial antitumor activity [24,25,26]. These findings provide a rationale for testing immunotherapy earlier in the definitive-treatment pathway for locally advanced disease.

Induction chemoimmunotherapy before CCRT may offer a specific therapeutic opportunity. Chemotherapy may release tumor antigens and reduce tumor burden, while PD-1 blockade may enhance antitumor immune activity before thoracic radiotherapy causes lymphopenia and inflammatory toxicity. Early studies integrating immunotherapy with definitive CCRT, including induction chemoimmunotherapy followed by CCRT, have reported encouraging activity and acceptable feasibility [27,28,29,30,31,32,33,34].

Most available studies have compared immunotherapy-containing strategies with CCRT alone or have reported single-arm outcomes. The more clinically precise question for patients already selected to receive induction therapy is whether immunotherapy adds benefit beyond induction chemotherapy itself. We therefore compared induction chemoimmunotherapy followed by CCRT (IOC-CCRT) with induction chemotherapy followed by CCRT (IC-CCRT) in a single-center retrospective cohort. We focused on survival, failure pattern, and treatment-related adverse events, while treating the findings as observational and hypothesis-generating.

## 2. Materials and Methods

### 2.1. Study Design and Patients

This retrospective cohort study included consecutive patients with locally advanced ESCC treated at Peking University Cancer Hospital & Institute between January 2017 and December 2024. Eligible patients were adults with histologically confirmed primary ESCC, locally advanced disease treated with definitive-intent CCRT, adequate baseline staging information obtained through a standardized pretreatment evaluation, including contrast-enhanced chest computed tomography (CT), esophagogastroduodenoscopy with biopsy (with endoscopic ultrasound [EUS] when feasible), barium esophagography, abdominal and cervical ultrasonography and/or CT, and positron emission tomography/computed tomography (PET/CT) when clinically indicated, available treatment details, and evaluable survival follow-up. Disease was staged according to the American Joint Committee on Cancer (AJCC) 8th edition. Patients with M1 disease limited to supraclavicular lymph node involvement were included according to the institutional treatment framework used for locally advanced ESCC. Patients were excluded if they had prior esophageal surgery, prior systemic therapy or radiotherapy for ESCC, distant visceral metastasis outside the defined locally advanced framework, another active malignancy within five years, or insufficient outcome data.

The study was approved by the Institutional Review Board of Peking University Cancer Hospital. The requirement for written informed consent was waived because of the retrospective design and use of de-identified clinical data. The study was conducted in accordance with the Declaration of Helsinki and reported according to the principles of the Strengthening the Reporting of Observational Studies in Epidemiology (STROBE) guideline [35].

### 2.2. Induction Therapy

Patients in both treatment groups received induction therapy every 3 weeks before definitive CCRT. The chemotherapy backbone consisted predominantly of a platinum-based doublet regimen, including paclitaxel or nab-paclitaxel combined with cisplatin or carboplatin.

Patients in the IC-CCRT group received induction chemotherapy alone, whereas those in the IOC-CCRT group received the same chemotherapy regimen combined with an anti-PD-1 monoclonal antibody. The PD-1 inhibitors included tislelizumab, camrelizumab, sintilimab, toripalimab and pembrolizumab, all of which were administered intravenously on day 1 of each 3-week treatment cycle according to the recommended dosing schedules.

Tumor response was assessed every two treatment cycles using contrast-enhanced chest computed tomography (CT), together with abdominal and cervical ultrasonography or CT, according to the Response Evaluation Criteria in Solid Tumors (RECIST), version 1.1 [36]. Patients who achieved disease control and remained unsuitable candidates for surgery subsequently proceeded to definitive CCRT.

### 2.3. Radiotherapy and Concurrent Chemotherapy

All patients received definitive radiotherapy using intensity-modulated radiotherapy (IMRT) or volumetric-modulated arc therapy (VMAT) with 6-MV photon beams, delivered five fractions per week. The prescribed radiation dose ranged from 50 to 60 Gy in 25–32 fractions. In most patients, the prescribed dose was 60 Gy to the planning gross tumor volume (PGTV) and 50.4 Gy to the planning target volume (PTV), both delivered in 28 fractions.

The gross tumor volume of the primary tumor (GTVp) was delineated according to contrast-enhanced CT, endoscopy with or without endoscopic ultrasonography (EUS), barium esophagography, and positron emission tomography/CT (PET/CT), when available. The gross tumor volume of metastatic lymph nodes (GTVn) included radiologically diagnosed or pathologically confirmed regional lymph nodes. Radiologically positive lymph nodes were identified by malignant imaging features, including a short-axis diameter of ≥10 mm in the mediastinal or abdominal regions, ≥5 mm in the paraesophageal, tracheoesophageal groove, or recurrent laryngeal nerve chain regions, abnormal fluorodeoxyglucose uptake on PET/CT, or imaging findings suggestive of malignancy such as central necrosis or ring enhancement.

The clinical target volume (CTV) comprised the GTVp with a 3 cm craniocaudal margin and a 0.5–0.8 cm radial margin, the GTVn with a 0.5–1.0 cm margin, and elective nodal regions according to the location of the primary tumor. The PGTV and PTV were generated by adding a uniform 0.5 cm three-dimensional margin to the GTVp/GTVn and CTV, respectively.

Concurrent chemotherapy consisted of a platinum-based doublet regimen administered either weekly or every 3 weeks throughout radiotherapy. For elderly patients or those unable to tolerate intravenous chemotherapy, particularly because of severe toxicities during induction therapy, oral S-1 (tegafur/gimeracil/oteracil) was permitted as an alternative concurrent regimen. No immunotherapy was administered during CCRT because of concerns regarding the potential increased risk of radiation pneumonitis.

### 2.4. Consolidation Therapy

Patients underwent their first evaluation 4–6 weeks after CCRT, including chest CT, esophagogram, abdominal and cervical ultrasonography and/or CT. For patients without disease progression or adverse events of grade > 2 (AEs), especially radiation pneumonitis, consolidation immunotherapy was given every 3 weeks for up to 2 years. Patients in both groups received TP-based consolidation chemotherapy (TP: paclitaxel or nab-paclitaxel combined with carboplatin or cisplatin).

### 2.5. Outcome Definitions and Assessment

Patients were scheduled for follow-up every 3 months during the first 2 years after treatment, every 6 months during the third and fourth years, and annually thereafter. Follow-up assessments routinely included physical examination, laboratory tests, contrast-enhanced chest CT, abdominal and cervical ultrasonography or CT, and barium esophagography. Patterns of treatment failure were categorized as locoregional recurrence (LRR), distant metastasis (DM), or mixed failure. LRR was defined as recurrence involving the primary esophageal tumor and/or regional lymph nodes, whereas DM referred to disease progression at distant anatomical sites. Mixed failure was defined as the coexistence of both locoregional recurrence and distant metastasis.

The primary endpoint was overall survival (OS), which was defined as the interval from treatment initiation to death from any cause. Treatment initiation was defined as the first day of induction therapy for patients in the IC-CCRT group and the IOC-CCRT group. Patients who were alive at the time of the analysis were censored at the date of their last follow-up.

Secondary endpoints comprised progression-free survival (PFS), patterns of failure, and treatment-related adverse events (TRAEs) graded according to the Common Terminology Criteria for Adverse Events (CTCAE), version 5.0. PFS was defined as the time from the corresponding treatment-initiation date to the first documented disease progression or death from any cause, whichever occurred first. ORR was defined as the proportion of patients who achieved a complete response (CR) or partial response (PR), whereas DCR was defined as the proportion who achieved CR, PR, or stable disease (SD).

### 2.6. Statistical Analysis

To minimize the impact of measured confounding associated with the nonrandomized study design, propensity score matching (PSM) was performed. Propensity scores were calculated using a multivariable logistic regression model with treatment group as the dependent variable and age, sex, body mass index (BMI), Eastern Cooperative Oncology Group performance status (ECOG PS), tumor location, clinical T stage, clinical N stage, clinical M stage, and radiation dose as covariates. Nearest-neighbor matching without replacement was conducted using a caliper width of 0.2 times the standard deviation of the logit of the propensity score. A variable-ratio matching strategy (2:1) was applied to maximize sample retention while maintaining covariate balance, yielding 141 patients in the IOC-CCRT group and 79 patients in the IC-CCRT group after matching. Covariate balance was evaluated using standardized mean differences (SMDs), with values < 0.10 considered indicative of acceptable balance.

Continuous variables were assessed for normality and are presented as mean ± standard deviation (SD) for normally distributed data or median (interquartile range [IQR]) for non-normally distributed data. Group comparisons were performed using the independent-samples *t* test or the Mann–Whitney U test, as appropriate. Categorical variables are expressed as frequencies and percentages and were compared using the χ^2^ test or Fisher’s exact test, as appropriate. Median follow-up was estimated using the reverse Kaplan–Meier method. Survival was estimated using the Kaplan–Meier method and compared with the log-rank test, while hazard ratios (HRs) and 95% confidence intervals (CIs) were calculated using Cox proportional hazards regression. Multivariable Cox regression analysis was performed in the full cohort to account for residual confounding and to identify independent prognostic factors. Variables with a *p* value < 0.10 in the univariable analysis or those considered clinically relevant based on established evidence were entered into the multivariable model. The proportional hazards assumption was evaluated using Schoenfeld residuals.

The cumulative incidences of locoregional recurrence (LRR), distant metastasis (DM), and mixed failure were estimated within a competing-risks framework, in which death without prior recurrence was treated as the competing event. Differences in cumulative incidence between the two treatment groups were compared using Gray’s test. Treatment-related adverse events (TRAEs) were summarized as the numbers and percentages of patients experiencing any-grade and grade 3–4 toxicities according to system-organ class, and comparisons between groups were performed using the χ^2^ test or Fisher’s exact test, as appropriate. All statistical analyses were two-sided, and statistical significance was defined as a *p* value < 0.05. Statistical analyses were conducted using R software (version 4.2; R Foundation for Statistical Computing, Vienna, Austria).

Programmed death-ligand 1 (PD-L1) expression was assessed using recorded combined positive score (CPS) values. Exploratory analyses were restricted to the 143 patients with available CPS data, representing 62.7% of the full IOC-CCRT cohort (143/228). Patients were classified into four mutually exclusive categories: CPS < 1, 1 to <5, 5 to <10, and ≥10; CPS ≥ 1 was defined as PD-L1 positive for this analysis. The boundaries of 1, 5, and 10 were informed by thresholds evaluated in a previous ESCC immunotherapy study [27]. These categories were used for exploratory analyses and were not considered validated predictive strata for the present treatment setting. OS and PFS were estimated using the Kaplan–Meier method and compared across the four CPS categories using global log-rank tests. *p* values were nominal and unadjusted for multiple testing, and no pairwise comparisons were performed. The supplementary analyses were performed using R version 4.5.2.

## 3. Results

### 3.1. Patient Population and Baseline Characteristics

The full cohort included 333 patients with locally advanced ESCC, including 228 patients in the IOC-CCRT group and 105 patients in the IC-CCRT group. Baseline characteristics are summarized in Table 1. Before propensity score matching, several clinically relevant differences were observed between groups. Patients in the IOC-CCRT group had a higher proportion of ECOG PS 0 than those in the IC-CCRT group (70.2% vs. 55.2%, *p* = 0.008) and were more likely to have tumors ≥5 cm in length (93.4% vs. 62.9%, *p* < 0.001). In addition, differences were observed in disease distribution, with a lower proportion of M1 disease in the IOC-CCRT group (14.9% vs. 27.6%, *p* = 0.010) and different AJCC stage distributions between groups (*p* = 0.008). Radiation dose category also differed before matching (*p* = 0.047), whereas age, sex, BMI category, tumor location, clinical T stage, clinical N stage, and concurrent chemotherapy regimen were comparable between groups.

The median number of induction cycles was 3 (IQR, 2–4) in the IOC-CCRT group and 2 (IQR, 2–3) in the IC-CCRT group (*p* < 0.001). The induction chemotherapy regimens and selected treatment-delivery characteristics are summarized in Table S1.

During definitive CCRT, radiotherapy parameters were comparable between groups. Most patients received VMAT (88.2%) or IMRT (11.8%), with a median PGTV dose of 60 Gy and a median of 28 fractions in both groups; the median PTV dose was 50.4 Gy. Concurrent chemotherapy was mainly based on taxane-platinum regimens, while S-1-based or other regimens were administered in selected patients. Detailed radiotherapy parameters and concurrent chemotherapy regimens are summarized in Table S1.

### 3.2. Survival Outcomes

**Figure 1:**
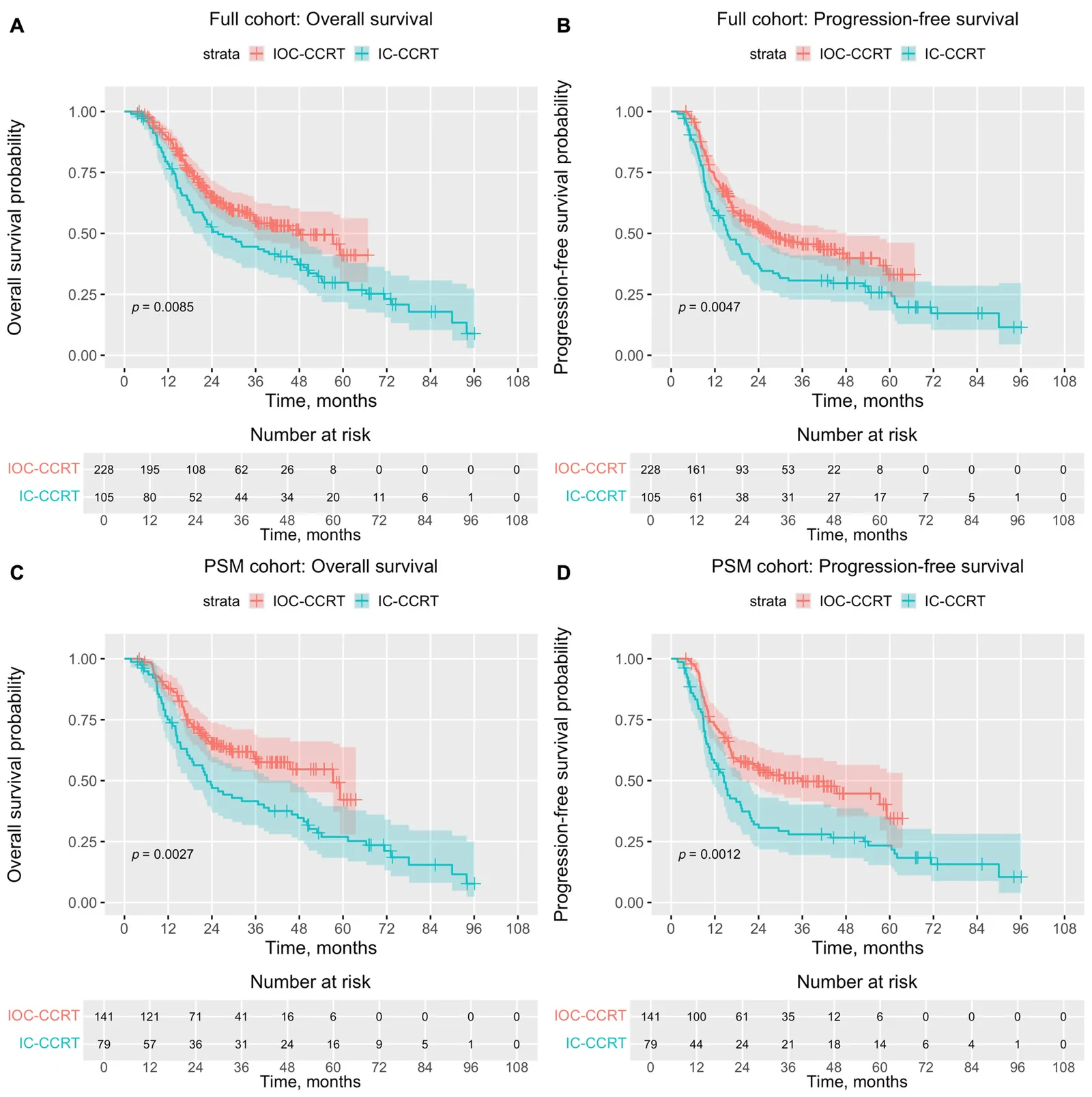
Kaplan–Meier curves for overall survival and progression-free survival in the IOC-CCRT and IC-CCRT groups before and after propensity score matching. (**A**) overall survival in the full cohort; (**B**) progression-free survival in the full cohort; (**C**) overall survival in the PSM cohort; (**D**) progression-free survival in the PSM cohort.

The median follow-up was 41.82 months (95% CI, 39.95–45.08 months) in the full cohort and 41.82 months (95% CI, 37.55–47.41 months) in the propensity score–matched cohort. IOC-CCRT was associated with significantly improved OS and PFS compared with IC-CCRT, with log-rank *p* values of 0.009 and 0.005, respectively. The 1-, 2-, and 3-year OS rates were 88.8%, 64.6%, and 55.1% in the IOC-CCRT group versus 78.5%, 52.7%, and 44.6% in the IC-CCRT group, respectively (Figure 1A). The corresponding PFS rates were 72.8%, 53.0%, and 45.6% versus 59.3%, 37.6%, and 30.7%, respectively (Figure 1B).

After propensity score matching, 220 patients were retained (141 in the IOC-CCRT group and 79 in the IC-CCRT group), with acceptable balance across measured covariates included in the propensity score model (Table 1). IOC-CCRT remained associated with significantly improved OS (*p* = 0.003), with 1-, 2-, and 3-year OS rates of 87.8%, 65.1%, and 59.0% versus 75.1%, 48.3%, and 41.6%, respectively (Figure 1C). PFS was also significantly prolonged with IOC-CCRT (*p* = 0.001), with 1-, 2-, and 3-year PFS rates of 72.6%, 55.2%, and 49.7% versus 57.3%, 32.0%, and 28.0%, respectively (Figure 1D). Kaplan–Meier survival curves are shown in Figure 1.

### 3.3. Failure Patterns

**Figure 2:**
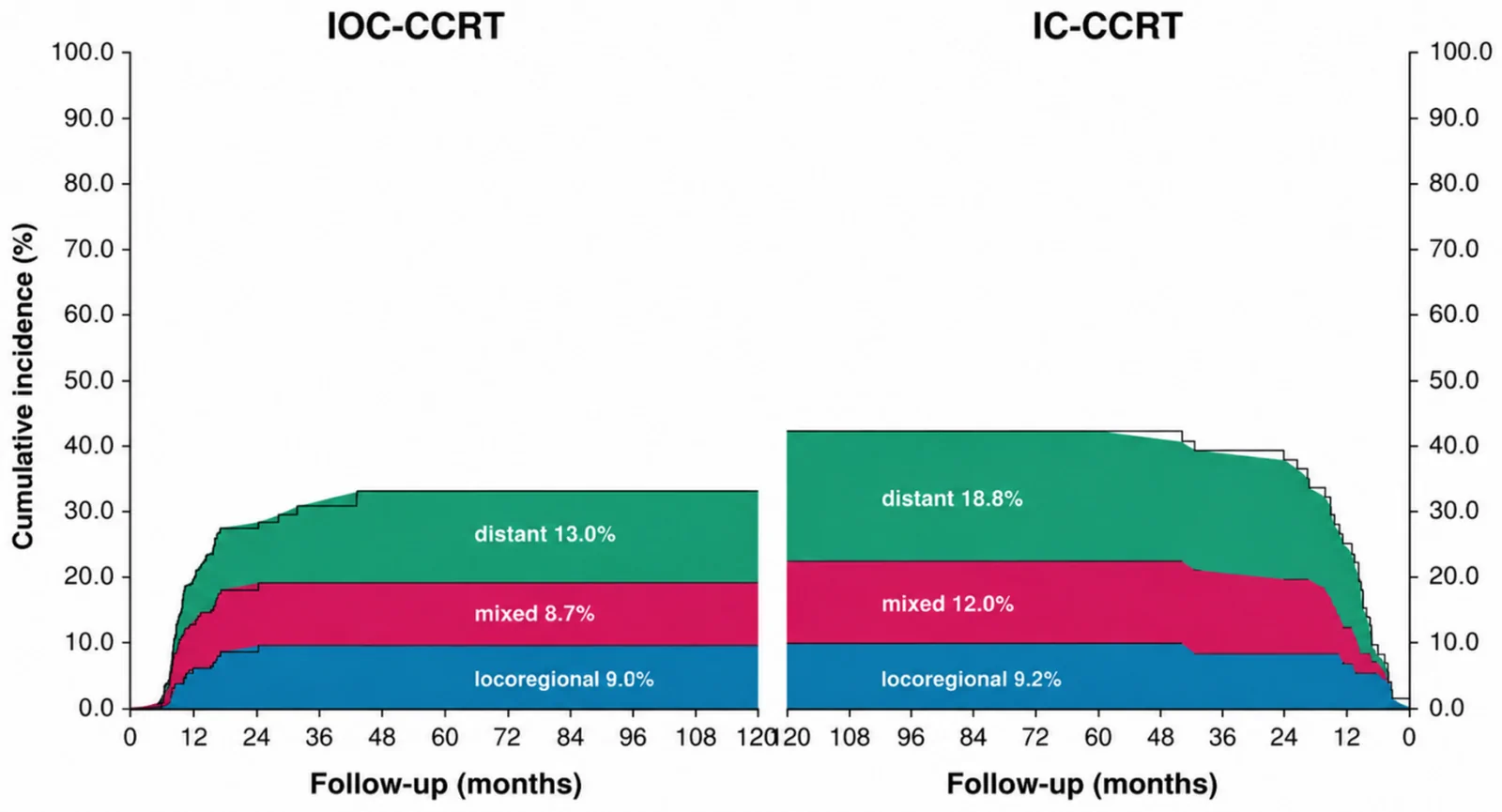
Cumulative incidence of locoregional recurrence, distant metastasis, and mixed failure in the IOC-CCRT and IC-CCRT groups after propensity score matching. Stacked areas represent mutually exclusive recurrence patterns; death without recurrence was treated as a competing event but is not displayed in the stacked recurrence plot.

Failure-pattern analysis in the matched cohort suggested numerically fewer total failures after IOC-CCRT, although the difference did not reach conventional statistical significance. Any recorded failure occurred in 39 of 141 IOC-CCRT patients (27.7%) and 31 of 79 IC-CCRT patients (39.2%; *p* = 0.077). Locoregional-only failure occurred in 12 IOC-CCRT patients (8.5%) and 8 IC-CCRT patients (10.1%; *p* = 0.689). Mixed failure occurred in 12 IOC-CCRT patients (8.5%) and 9 IC-CCRT patients (11.4%; *p* = 0.485). Distant-only metastasis occurred in 15 IOC-CCRT patients (10.6%) and 14 IC-CCRT patients (17.7%; *p* = 0.136). The detailed failure distribution is shown in Table 2, and the cumulative incidence visualization is shown in Figure 2.

### 3.4. Univariable and Multivariable Analyses

In the full cohort before propensity score matching, univariable Cox analysis showed that IOC-CCRT was associated with improved OS compared with IC-CCRT (HR, 0.66; 95% CI, 0.48–0.90; *p* = 0.009). In the multivariable model, this association was attenuated and no longer statistically significant (HR, 0.82; 95% CI, 0.58–1.14; *p* = 0.234). PGTV dose ≥60 Gy showed a favorable association with OS (HR, 0.73; 95% CI, 0.52–1.03; *p* = 0.073), while consolidation immunotherapy was independently associated with improved OS (HR, 0.40; 95% CI, 0.25–0.66; *p* < 0.001).

For PFS, univariable Cox analysis showed that IOC-CCRT was associated with improved PFS compared with IC-CCRT (HR, 0.66; 95% CI, 0.50–0.88; *p* = 0.005). After multivariable adjustment, IOC-CCRT remained favorably associated with PFS, although the association did not reach statistical significance (HR, 0.75; 95% CI, 0.55–1.03; *p* = 0.075). PGTV dose ≥60 Gy (HR, 0.58; 95% CI, 0.43–0.79; *p* < 0.001) and consolidation immunotherapy (HR, 0.60; 95% CI, 0.41–0.90; *p* = 0.014) were independently associated with improved PFS. Detailed Cox regression results for OS and PFS are presented in Table 3a,b, with additional details provided in Supplementary Table S2a,b.

### 3.5. Treatment-Related Adverse Events

TRAEs were generally manageable in both treatment groups. After PSM, hematologic toxicities remained the most common any-grade TRAEs. Bone marrow suppression was comparable between the IOC-CCRT and IC-CCRT groups (95.0% vs. 92.4%; *p* = 0.552). Neutropenia was more frequent in the IOC-CCRT group (66.7% vs. 49.4%; *p* = 0.012), whereas anemia (57.4% vs. 46.8%; *p* = 0.130), leukopenia (85.1% vs. 87.3%; *p* = 0.648), and thrombocytopenia (48.2% vs. 39.2%; *p* = 0.199) were comparable between the groups. Among nonhematologic TRAEs, fatigue (42.6% vs. 25.3%; *p* = 0.011), nausea (40.4% vs. 24.1%; *p* = 0.014), and radiation esophagitis (63.1% vs. 34.2%; *p* < 0.001) were more frequent in the IOC-CCRT group.

Grade 3 or higher hematologic toxicities were the predominant severe TRAEs after PSM. Neutropenia was more frequent in the IOC-CCRT group (31.9% vs. 16.5%; *p* = 0.013), whereas leukopenia (27.0% vs. 21.5%; *p* = 0.372), and thrombocytopenia (12.1% vs. 5.1%; *p* = 0.090) were comparable between the groups. Grade 3 or higher nonhematologic TRAEs were uncommon, although radiation esophagitis was more frequent in the IOC-CCRT group (6.4% vs. 0%; *p* = 0.028). Detailed results are presented in Table 4.

### 3.6. Exploratory Analysis by PD-L1 CPS Category

Among the full IOC-CCRT cohort before propensity score matching, 143/228 (62.7%) had available CPS data and were included in this exploratory analysis. The CPS categories <1, 1 to <5, 5 to <10, and ≥10 included 57 (39.9%), 21 (14.7%), 26 (18.2%), and 39 (27.3%) patients, respectively. Overall, 86/143 patients (60.1%) were classified as PD-L1 positive using CPS ≥1.

PFS differed across the four CPS categories (*p* = 0.022), whereas the corresponding difference in OS was not statistically significant (*p* = 0.153; Figure S1A,B). The 36-month OS estimates were 49.7%, 60.8%, 50.8%, and 80.1% for CPS <1, 1 to <5, 5 to <10, and ≥10, respectively; the corresponding PFS estimates were 35.3%, 41.7%, 47.3%, and 72.5%.

## 4. Discussion

To our knowledge, this study represents one of the largest propensity score-matched comparative studies evaluating the addition of immunotherapy to induction chemotherapy before definitive CCRT in patients with locally advanced ESCC. After adjustment for baseline imbalances, IOC-CCRT was associated with significantly improved OS and PFS compared with IC-CCRT.

The present comparison is clinically relevant because both treatment groups received induction therapy before definitive CCRT, making the choice of comparison group particularly important for interpreting these findings. Previous studies have reported inconsistent benefits of induction chemotherapy before definitive chemoradiotherapy in locally advanced ESCC [11,12,13,14,15,16,17]. In the present study, both groups received induction systemic therapy followed by definitive CCRT, allowing the incremental contribution of immunotherapy to be evaluated more directly, rather than the effect of induction treatment itself. Therefore, the improved outcomes observed with IOC-CCRT may reflect the additional therapeutic effect of immune checkpoint inhibition; however, direct evidence comparing IOC-CCRT with IC-CCRT remains limited.

Most published studies have compared induction chemoimmunotherapy followed by definitive radiotherapy or chemoradiotherapy with CCRT alone or historical controls rather than chemotherapy-only induction [18,19,20,21,22,23,24,25,26,27,28,29,30,31,32,33,34]. Despite these differences in study design, most studies have consistently suggested favorable survival outcomes following the incorporation of immunotherapy into induction treatment.

Accumulating evidence has shown that PD-1 blockade plus chemotherapy improves outcomes in advanced ESCC, whereas neoadjuvant chemoimmunotherapy has demonstrated encouraging activity in operable disease [18,19,20,21,22,23,24,25,26]. Early definitive-treatment studies have also supported the feasibility of integrating immunotherapy with chemoradiotherapy-based strategies [27,28,29,30,31,32,33,34]. Our study extends this literature by using IC-CCRT as the comparator, which is closer to the clinical decision faced when induction treatment is already being considered.

Several biological mechanisms may explain these observations. Chemotherapy can promote antigen release and cytoreduction, while PD-1 blockade may restore antitumor immune function. Delivering immunotherapy before thoracic radiotherapy may also avoid part of the immune suppression associated with radiation-related lymphopenia. Translational studies of immune normalization, vascular normalization, and PD-L1 regulation provide a plausible framework for interaction between immunotherapy and radiotherapy response [37,38,39,40]. These mechanisms were not directly tested in this retrospective study.

Analysis of recurrence patterns provided additional insight into the observed survival benefit. IOC-CCRT showed numerically fewer total failures and distant-only failures, although the differences did not reach statistical significance. The survival association should therefore not be attributed to one failure pathway alone. It may reflect the combined effects of improved systemic control, enhanced locoregional control, treatment tolerance, and residual patient heterogeneity.

The safety profile should also be considered when interpreting the potential clinical benefit of IOC-CCRT. In both the full and matched cohorts, hematologic toxicities were the predominant TRAEs. IOC-CCRT was associated with higher incidences of grade ≥ 3 bone marrow suppression and neutropenia, as well as more frequent anemia, hepatic enzyme elevation, peripheral neuropathy, and radiation esophagitis. Notably, prophylactic granulocyte colony-stimulating factor (G-CSF) was used less frequently in the IOC-CCRT group than in the IC-CCRT group (27/141 [19.1%] vs. 43/79 [54.4%]), which may have contributed to the higher incidence of severe hematologic toxicity observed with IOC-CCRT. Most severe nonhematologic toxicities remained uncommon, although grade 3 or higher radiation esophagitis occurred more frequently in the IOC-CCRT group. These findings suggest that IOC-CCRT is feasible in appropriately selected patients with careful toxicity monitoring and supportive management. Nevertheless, these toxicities should be considered alongside the potential survival benefit of IOC-CCRT.

The exploratory CPS-stratified analysis identified differences in PFS across the four PD-L1 CPS categories within the IOC-CCRT analysis subset (*p* = 0.022), whereas the corresponding difference in OS did not reach statistical significance (*p* = 0.153). Patients with CPS ≥ 10 generally had more favorable survival estimates, although the global comparisons do not establish significant differences between individual categories or a graded relationship between CPS and survival. These findings are compatible with the potential relevance of PD-L1 expression to outcomes following chemoimmunotherapy. In KEYNOTE-590, pembrolizumab plus chemotherapy improved survival in advanced ESCC, including the CPS ≥ 10 population [18]. However, exploratory analyses of JUPITER-06 also demonstrated benefit in patients with low PD-L1 expression [23], indicating that low expression does not necessarily preclude benefit. These studies provide context for our observations, but their findings cannot be directly extrapolated to induction chemoimmunotherapy followed by definitive CCRT.

Our results should therefore be considered hypothesis-generating. An association between CPS and survival within a single treatment cohort cannot distinguish a prognostic association from a predictive effect on the relative benefit of IOC-CCRT versus IC-CCRT. In the IC-CCRT cohort, CPS results were available for 67 of 105 patients, all of whom had CPS < 1. This incomplete ascertainment and absence of CPS-positive patients among those tested precluded a meaningful treatment-by-CPS interaction analysis across the CPS categories. Consequently, biomarker-related treatment selection may have contributed to the observed between-group survival differences and cannot be excluded by the within-cohort analyses. Small subgroup sizes and declining numbers at risk further limit the precision of long-term survival estimates.

Several limitations should be considered when interpreting these findings. This was a single-center retrospective study, and propensity score methods cannot eliminate unmeasured confounding [41]. Missing factors may include PD-L1 expression, nutritional status, sarcopenia, inflammatory markers, comorbidity burden, treatment access, and physician preference. Treatment was heterogeneous, including different PD-1 inhibitors, chemotherapy backbones, induction cycles, radiotherapy details, and consolidation practices. Follow-up maturity may also differ because immunotherapy was adopted later in routine care. Recurrence and toxicity outcomes were retrospectively abstracted and may underreport delayed or low-grade events.

The data support further study rather than immediate practice change. Prospective trials should compare chemoimmunotherapy-based induction with chemotherapy-only induction before CCRT, standardize systemic and radiation regimens, and stratify by tumor burden and nodal extent. Biomarkers of immune activity, nutrition, inflammation, and minimal residual disease may help identify patients most likely to benefit. Nevertheless, these findings should be interpreted cautiously given the retrospective nature of this study, and prospective randomized studies are warranted to further validate the role of chemoimmunotherapy-based induction before definitive CCRT.

## 5. Conclusions

In this propensity score-matched retrospective study of patients with locally advanced ESCC, IOC-CCRT was associated with longer OS and PFS than IC-CCRT in Kaplan–Meier analyses of both the full and matched cohorts. By comparing two induction-based treatment strategies before definitive CCRT, this study provides clinically relevant evidence regarding the potential value of incorporating immunotherapy into induction treatment. These findings support further evaluation of induction chemoimmunotherapy within the definitive treatment pathway for locally advanced ESCC. Careful patient selection and coordinated systemic and radiation treatment are important considerations when developing this approach. Prospective studies incorporating standardized treatment protocols and biomarker assessment are warranted to validate the observed survival associations, identify patients most likely to benefit, and refine the role of induction chemoimmunotherapy before definitive CCRT.

## Figures and Tables

**Table 1:** Patient demographics and baseline characteristics.

Characteristics		Before Propensity Score Matching, *n* (%)			After Propensity Score Matching, *n* (%)		
		IOC-CCRT Group	IC-CCRT Group	*p* Value	IOC-CCRT Group	IC-CCRT Group	*p* Value
		(*n* = 228)	(*n* = 105)		(*n* = 141)	(*n* = 79)	
Age (years) Mean (SD)		62.55 (8.21)	63.95 (8.08)	0.144	63.27 (8.34)	63.22 (8.41)	0.963
Sex	Male	197 (86.4)	91 (86.7)	0.948	122 (86.5)	69 (87.3)	0.864
	Female	31 (13.6)	14 (13.3)		19 (13.5)	10 (12.7)	
BMI	<25	186 (81.6)	85 (81.0)	0.891	113 (80.1)	65 (82.3)	0.699
	≥25	42 (18.4)	20 (19.0)		28 (19.9)	14 (17.7)	
ECOG	0	160 (70.2)	58 (55.2)	0.008	88 (62.4)	49 (62.0)	0.955
	1–2	68 (29.8)	47 (44.8)		53 (37.6)	30 (38.0)	
Tumor length (cm)	<5	15 (6.6)	39 (37.1)	<0.001	15 (10.6)	14 (17.7)	0.136
	≥5	213 (93.4)	66 (62.9)		126 (89.4)	65 (82.3)	
Tumor location	Upper segment	91 (39.9)	39 (37.1)	0.685	47 (33.3)	27 (34.2)	0.989
	Middle segment	82 (36.0)	36 (34.3)		50 (35.5)	28 (35.4)	
	Lower segment	55 (24.1)	30 (28.6)		44 (31.2)	24 (30.4)	
T stage	T1-2	22 (9.6)	9 (8.6)	0.088	12 (8.5)	5 (6.3)	0.838
	T3	119 (52.2)	68 (64.8)		90 (63.8)	51 (64.6)	
	T4	87 (38.2)	28 (26.7)		39 (27.7)	23 (29.1)	
N stage	N0-1	56 (24.6)	31 (29.5)	0.338	32 (22.7)	23 (29.1)	0.292
	N2-3	172 (75.4)	74 (70.5)		109 (77.3)	56 (70.9)	
M stage	M0	194 (85.1)	76 (72.4)	0.010	112 (79.4)	57 (72.2)	0.307
	M1	34 (14.9)	29 (27.6)		29 (20.6)	22 (27.8)	
AJCC stage	III	106 (46.5)	49 (46.7)	0.008	70 (49.6)	36 (45.6)	0.469
	IVA	88 (38.6)	27 (25.7)		42 (29.8)	21 (26.6)	
	IVB	34 (14.9)	29 (27.6)		29 (20.6)	22 (27.8)	
Radiation dose (Gy)	<60	79 (34.6)	25 (23.8)	0.047	42 (29.8)	23 (29.1)	0.916
	≥60	149 (65.4)	80 (76.2)		99 (70.2)	56 (70.9)	
Concurrent chemotherapy regimen	TP-based/Taxane-platinum	202 (88.6)	87 (82.9)	0.151	126 (89.4)	66 (83.5)	0.214
	S-1/other	26 (11.4)	18 (17.1)		15 (10.6)	13 (16.5)	

**Table 2:** Failure patterns after treatment in the propensity score-matched cohort.

Failure Pattern	Overall	IC-CCRT (*n* = 79)	IOC-CCRT (*n* = 141)	*p* Value
No failure	150	48	102	0.077
Total failure	70	31	39	0.077
Locoregional only	20	8	12	0.689
Esophageal recurrence only	10	3	7	1.000
Nodal recurrence only	8	5	3	0.139
Esophageal and nodal recurrence	2	0	2	0.538
Locoregional with distant metastasis	21	9	12	0.485
Esophageal recurrence with distant metastasis	6	3	3	0.669
Nodal recurrence with distant metastasis	12	5	7	0.759
Esophageal and nodal recurrence with distant metastasis	2	0	2	0.538
Unclassified locoregional recurrence with distant metastasis	1	1	0	0.359
Distant metastasis only	29	14	15	0.136
Lung	7	4	3	0.254
Liver	3	2	1	0.293
Bone	3	1	2	1.000
Brain	5	1	4	0.657
Cervical/supraclavicular lymph nodes	5	3	2	0.353
Multiple metastatic sites	4	1	3	1.000
Other sites	2	2	0	0.128

**Table 3:** (**a**) Univariable and multivariable Cox regression analyses for overall survival in the full cohort. (**b**) Univariable and multivariable Cox regression analyses for progression-free survival in the full cohort.

(a)				
Variables	Univariable Analysis		Multivariable Analysis	
	HR (95% CI)	*p* Value	HR (95% CI)	*p* Value
Treatment group (IOC-CCRT vs. IC-CCRT)	0.66 (0.48–0.90)	0.009	0.82 (0.58–1.14)	0.234
Clinical T stage (T3 vs. T1-2)	1.26 (0.73–2.17)	0.405	—	—
Clinical T stage (T4 vs. T1-2)	1.10 (0.62–1.95)	0.744	—	—
Clinical N stage (N2-3 vs. N0-1)	1.42 (0.98–2.06)	0.062	1.36 (0.93–1.97)	0.112
Clinical M stage (M1 vs. M0)	0.81 (0.52–1.24)	0.332	—	—
PGTV dose (≥60 vs. <60 Gy)	0.69 (0.49–0.95)	0.025	0.73 (0.52–1.03)	0.073
Coronary heart disease (yes vs. no)	0.44 (0.19–0.99)	0.047	0.42 (0.18–0.96)	0.039
Consolidation immunotherapy (yes vs. no)	0.38 (0.23–0.60)	<0.001	0.40 (0.25–0.66)	<0.001
Consolidation immunotherapy cycles	0.91 (0.87–0.96)	<0.001	—	—
(**b**)				
**Variables**	**Univariable Analysis**		**Multivariable Analysis**	
	**HR (95% CI)**	***p* Value**	**HR (95% CI)**	***p* Value**
Treatment group (IOC-CCRT vs. IC-CCRT)	0.66 (0.50–0.88)	0.005	0.75 (0.55–1.03)	0.075
Clinical T stage (T3 vs. T1-2)	1.61 (0.95–2.71)	0.074	1.60 (0.95–2.70)	0.077
Clinical T stage (T4 vs. T1-2)	1.26 (0.73–2.18)	0.409	1.20 (0.69–2.09)	0.517
Clinical N stage (N2-3 vs. N0-1)	1.31 (0.94–1.83)	0.109	—	—
Clinical M stage (M1 vs. M0)	0.83 (0.56–1.21)	0.330	—	—
PGTV dose (≥60 vs. <60 Gy)	0.61 (0.45–0.82)	<0.001	0.58 (0.43–0.79)	<0.001
Coronary heart disease (yes vs. no)	0.60 (0.31–1.18)	0.141	—	—
Consolidation immunotherapy (yes vs. no)	0.53 (0.36–0.76)	<0.001	0.60 (0.41–0.90)	0.014
Consolidation immunotherapy cycles	0.93 (0.89–0.96)	<0.001	—	—

Notes: (**a**) Cox regression analyses were performed in the full cohort (*N* = 333). (**b**) Cox regression analyses were performed in the full cohort (*N* = 333). Abbreviations: CI, confidence interval; HR, hazard ratio; IC-CCRT, induction chemotherapy followed by concurrent chemoradiotherapy; IOC-CCRT, induction chemoimmunotherapy followed by concurrent chemoradiotherapy; PGTV, planning gross tumor volume.

**Table 4:** Overall treatment-related adverse events in the propensity score-matched cohort.

Adverse Event	Any Grade, *n* (%)			Grade ≥ 3, *n* (%)		
	IOC-CCRT Group	IC-CCRT Group	*p* Value	IOC-CCRT Group	IC-CCRT Group	*p* Value
	(*n* = 141)	(*n* = 79)		(*n* = 141)	(*n* = 79)	
Bone marrow suppression	134 (95.0)	73 (92.4)	0.552	61 (43.3)	29 (36.7)	0.344
Leukopenia	120 (85.1)	69 (87.3)	0.648	38 (27.0)	17 (21.5)	0.372
Neutropenia	94 (66.7)	39 (49.4)	0.012	45 (31.9)	13 (16.5)	0.013
Anemia	81 (57.4)	37 (46.8)	0.130	5 (3.5)	0 (0.0)	0.163
Thrombocytopenia	68 (48.2)	31 (39.2)	0.199	17 (12.1)	4 (5.1)	0.090
Elevated ALT/AST	33 (23.4)	9 (11.4)	0.030	2 (1.4)	0 (0.0)	0.538
Hyperbilirubinemia	14 (9.9)	6 (7.6)	0.563	0 (0.0)	0 (0.0)	—
Fatigue	60 (42.6)	20 (25.3)	0.011	1 (0.7)	0 (0.0)	1.000
Nausea	57 (40.4)	19 (24.1)	0.014	3 (2.1)	0 (0.0)	0.555
Decreased appetite	39 (27.7)	2 (2.5)	<0.001	1 (0.7)	0 (0.0)	1.000
Rash	7 (5.0)	0 (0.0)	0.051	1 (0.7)	0 (0.0)	1.000
Peripheral neuropathy	31 (22.0)	0 (0.0)	<0.001	0 (0.0)	0 (0.0)	—
Immune-related hepatitis	2 (1.4)	0 (0.0)	0.538	1 (0.7)	0 (0.0)	1.000
Radiation esophagitis	89 (63.1)	27 (34.2)	<0.001	9 (6.4)	0 (0.0)	0.028
Cough	19 (13.5)	7 (8.9)	0.309	0 (0.0)	0 (0.0)	—
Radiation pneumonitis	5 (3.5)	1 (1.3)	0.423	0 (0.0)	0 (0.0)	–
Radiation dermatitis	14 (9.9)	2 (2.5)	0.043	0 (0.0)	0 (0.0)	—

Abbreviations: IC-CCRT, induction chemotherapy followed by concurrent chemoradiotherapy; IOC-CCRT, induction chemoimmunotherapy followed by concurrent chemoradiotherapy; ALT, alanine aminotransferase; AST, aspartate aminotransferase. *p* values were calculated using the χ^2^ test or Fisher’s exact test, as appropriate.

## Data Availability

The data presented in this study are available from the corresponding author upon reasonable request. The data are not publicly available due to patient privacy and institutional ethical restrictions.

## Supplementary Materials

The following supporting information can be downloaded at https://www.mdpi.com/article/10.3390/jcm15208121/s1, Figure S1: OS and PFS by recorded PD-L1 CPS category in the 143-patient IOC-CCRT analysis subset; Table S1: Baseline characteristics and selected treatment-delivery details in the full cohort (*N* = 333); Table S2a: Cox regression analyses for overall survival in the full cohort; Table S2b: Cox regression analyses for progression-free survival in the full cohort.
