# Supplementary material for: Induction Chemoimmunotherapy Versus Induction Chemotherapy Followed by Concurrent Chemoradiotherapy for Locally Advanced Esophageal Squamous Cell Carcinoma: A Propensity Score-Matched Study
Source: J Clin Med. 2026 Oct 19;15(20):8121. doi: 10.3390/jcm15208121 (PMC13706056; doi:10.3390/jcm15208121)
Supplement: Supplementary file 1 [file jcm-15-08121-s001.zip › Supplementary_Tables_S1_S2.pdf]

Supplementary Table S1. Baseline characteristics and selected treatment-delivery details in the full cohort (N = 333).

Characteristics	Overall N = 333, n (%)	IOC-CCRT group N = 228, n (%)	IC-CCRT group N = 105, n (%)	P value
**Sex**				**0.948**
Male	288 (86.5)	197 (86.4)	91 (86.7)	
Female	45 (13.5)	31 (13.6)	14 (13.3)	
Age (years), median (IQR)	63 (57-69)	63 (57-69)	64 (59-70)	0.136
**ECOG PS**				**0.008**
0	218 (65.5)	160 (70.2)	58 (55.2)	
1	115 (34.5)	68 (29.8)	47 (44.8)	
2	0 (0.0)	0 (0.0)	0 (0.0)	
**Smoking history**				**0.869**
No	90 (27.0)	61 (26.8)	29 (27.6)	
Yes	243 (73.0)	167 (73.2)	76 (72.4)	
**Alcohol history**				**0.232**
No	124 (37.2)	80 (35.1)	44 (41.9)	
Yes	209 (62.8)	148 (64.9)	61 (58.1)	
**Tumor length ≥5 cm**				**<0.001**
<5 cm	54 (16.2)	15 (6.6)	39 (37.1)	
≥5 cm	279 (83.8)	213 (93.4)	66 (62.9)	
**Tumor location**				**0.685**
Cervical/upper thoracic	130 (39.0)	91 (39.9)	39 (37.1)	
Middle thoracic	118 (35.4)	82 (36.0)	36 (34.3)	
Lower thoracic/GEJ	85 (25.5)	55 (24.1)	30 (28.6)	
**cT stage**				**0.088**
cT1-2	31 (9.3)	22 (9.6)	9 (8.6)	
cT3	187 (56.2)	119 (52.2)	68 (64.8)	
cT4	115 (34.5)	87 (38.2)	28 (26.7)	
**cN stage**				**0.338**
cN0–1	87 (26.1)	56 (24.6)	31 (29.5)	
cN2–3	246 (73.9)	172 (75.4)	74 (70.5)	
**cM stage**				**0.010**
cM0	271 (81.4)	194 (85.1)	77 (73.3)	
cM1	62 (18.6)	34 (14.9)	28 (26.7)	

Characteristics	Overall<br>N = 333, n (%)	IOC-CCRT group<br>N = 228, n (%)	IC-CCRT group<br>N = 105, n (%)	P value
**Stage (AJCC 8th)**				**0.008**
III	155 (46.5)	106 (46.5)	49 (46.7)	
IVA	115 (34.5)	88 (38.6)	27 (25.7)	
IVB	63 (18.9)	34 (14.9)	29 (27.6)	
**Induction chemotherapy regimen**				**<0.001**
Taxane/platinum	324 (97.3)	228 (100.0)	96 (91.4)	
S-1	1 (0.3)	0 (0.0)	1 (1.0)	
Others	8 (2.4)	0 (0.0)	8 (7.6)	
Cycles of induction therapy, median (IQR)	2 (2–4)	3 (2–4)	2 (2–3)	<0.001
**PGTV dose ≥60 Gy**				**0.047**
No	104 (31.2)	79 (34.6)	25 (23.8)	
Yes	229 (68.8)	149 (65.4)	80 (76.2)	
Radiation dose (Gy), median (IQR)	60 (56-60)	60 (56-60)	60 (60-60)	0.201
**Concurrent chemotherapy regimen**				**0.151**
TP-based/Taxane-platinum	289 (86.8)	202 (88.6)	87 (82.9)	
S-1/other	44 (13.2)	26 (11.4)	18 (17.1)	
Concurrent chemotherapy cycles, median (IQR)	2 (2-2)	2 (1-2)	2 (2-4)	<0.001
**Consolidation therapy**				**<0.001**
Yes	98 (29.4)	97 (42.5)	1 (1.0)	
No	235 (70.6)	131 (57.5)	104 (99.0)	
Cycles of consolidation therapy, median (IQR)	2 (1-4)	2 (1-4.8)	2 (2-2)	1.000

Abbreviations: AJCC, American Joint Committee on Cancer; CCRT, concurrent chemoradiotherapy; ECOG PS, Eastern Cooperative Oncology Group performance status; GEJ, gastroesophageal junction; IC-CCRT, induction chemotherapy followed by concurrent chemoradiotherapy; IOC-CCRT, induction chemoimmunotherapy followed by concurrent chemoradiotherapy; IQR, interquartile range; PGTV, planning gross tumor volume.

Continuous variables are presented as median (IQR) and compared using the Mann–Whitney U test. Categorical variables are presented as n (%) and compared using the chi-square test or Fisher exact test, as appropriate.

Supplementary Table 2a. Cox regression analyses for overall survival(N = 333).

Variables	Univariable analysis		Multivariable analysis	
	HR (95% CI)	P value	HR (95% CI)	P value
Treatment group (IOC-CCRT vs IC-CCRT)	0.66 (0.48-0.90)	0.009	0.82 (0.58-1.14)	0.234
Sex (Female vs Male)	0.75 (0.45-1.24)	0.263		
Age, years	1.00 (0.98-1.01)	0.687		
ECOG performance status (1 vs 0)	1.08 (0.79-1.47)	0.648		
BMI (≥25 vs <25 kg/m²)	0.87 (0.58-1.29)	0.474		
Tumor length (≥5 vs <5 cm)	1.06 (0.72-1.57)	0.751		
Tumor location (middle vs cervical/upper)	1.15 (0.80-1.65)	0.439		
Tumor location (lower/GEJ vs cervical/upper)	1.42 (0.97-2.09)	0.070		
Clinical T stage (T3 vs T1-2)	1.26 (0.73-2.17)	0.405		
Clinical T stage (T4 vs T1-2)	1.10 (0.62-1.95)	0.744		
Clinical N stage (N2-3 vs N0-1)	1.42 (0.98-2.06)	0.062	1.36 (0.93-1.97)	0.112
Clinical M stage (M1 vs M0)	0.81 (0.52-1.24)	0.332		
PGTV dose (≥60 vs <60 Gy)	0.69 (0.49-0.95)	0.025	0.73 (0.52-1.03)	0.073
Smoking history (yes vs no)	1.08 (0.76-1.53)	0.662		
Alcohol use (yes vs no)	1.04 (0.76-1.42)	0.814		
Coronary heart disease (yes vs no)	0.44 (0.19-0.99)	0.047	0.42 (0.18-0.96)	0.039
Hypertension (yes vs no)	1.00 (0.72-1.40)	0.998		
Diabetes (yes vs no)	0.76 (0.44-1.31)	0.323		
COPD (yes vs no)	0.41 (0.10-1.69)	0.218		
Concurrent chemotherapy (S-1 vs TP)	1.00 (0.62-1.60)	0.994		
Concurrent chemotherapy (others vs TP)	1.76 (0.95-3.27)	0.072		
Concurrent chemotherapy cycles	1.02 (0.91-1.14)	0.734		
Consolidation chemotherapy (TP vs none)	0.42 (0.06-3.01)	0.389		
Consolidation chemotherapy (S-1 vs none)	0.93 (0.34-2.51)	0.887		
Consolidation chemotherapy (others vs none)	0.19 (0.03-1.34)	0.095		
Consolidation chemotherapy cycles	0.80 (0.59-1.08)	0.143		
Consolidation immunotherapy (yes vs no)	0.38 (0.23-0.60)	<0.001	0.40 (0.25-0.66)	<0.001
Consolidation immunotherapy cycles	0.91 (0.87-0.96)	<0.001		

Abbreviations: BMI, body mass index; CI, confidence interval; COPD, chronic obstructive pulmonary disease; ECOG, Eastern Cooperative Oncology Group; GEJ, gastroesophageal junction; HR, hazard ratio; IC-CCRT, induction chemotherapy followed by concurrent chemoradiotherapy; IOC-CCRT, induction chemoimmunotherapy followed by concurrent chemoradiotherapy; OS, overall survival; PGTV, planning gross tumor volume; TP, taxane plus platinum.

Supplementary Table 2b. Cox regression analyses for progression-free survival(N = 333).

Variables	Univariable analysis		Multivariable analysis	
	HR (95% CI)	P value	HR (95% CI)	P value
Treatment group (IOC-CCRT vs IC-CCRT)	0.66 (0.50-0.88)	0.005	0.75 (0.55-1.03)	0.075
Sex (Female vs Male)	0.78 (0.50-1.22)	0.274		
Age, years	0.99 (0.97-1.01)	0.257		
ECOG performance status (1 vs 0)	1.08 (0.81-1.44)	0.594		
BMI (≥25 vs <25 kg/m²)	0.81 (0.56-1.18)	0.274		
Tumor length (≥5 vs <5 cm)	1.03 (0.72-1.46)	0.891		
Tumor location (middle vs cervical/upper)	1.08 (0.78-1.51)	0.631		
Tumor location (lower/GEJ vs cervical/upper)	1.28 (0.91-1.82)	0.161		
Clinical T stage (T3 vs T1-2)	1.61 (0.95-2.71)	0.074	1.60 (0.95-2.70)	0.077
Clinical T stage (T4 vs T1-2)	1.26 (0.73-2.18)	0.409	1.20 (0.69-2.09)	0.517
Clinical N stage (N2-3 vs N0-1)	1.31 (0.94-1.83)	0.109		
Clinical M stage (M1 vs M0)	0.83 (0.56-1.21)	0.330		
PGTV dose (≥60 vs <60 Gy)	0.61 (0.45-0.82)	<0.001	0.58 (0.43-0.79)	<0.001
Smoking history (yes vs no)	1.04 (0.76-1.43)	0.804		
Alcohol use (yes vs no)	1.05 (0.79-1.40)	0.732		
Coronary heart disease (yes vs no)	0.60 (0.31-1.18)	0.141		
Hypertension (yes vs no)	0.99 (0.73-1.35)	0.968		
Diabetes (yes vs no)	0.68 (0.40-1.15)	0.148		
COPD (yes vs no)	0.45 (0.11-1.83)	0.261		
Concurrent chemotherapy (S-1 vs TP)	1.15 (0.76-1.76)	0.506		
Concurrent chemotherapy (others vs TP)	1.55 (0.86-2.79)	0.145		
Concurrent chemotherapy cycles	1.03 (0.93-1.14)	0.582		
Consolidation chemotherapy (TP vs none)	0.65 (0.16-2.63)	0.547		
Consolidation chemotherapy (S-1 vs none)	1.23 (0.54-2.77)	0.620		
Consolidation chemotherapy (others vs none)	0.53 (0.17-1.68)	0.283		
Consolidation chemotherapy cycles	0.92 (0.78-1.07)	0.282		
Consolidation immunotherapy (yes vs no)	0.53 (0.36-0.76)	<0.001	0.60 (0.41-0.90)	0.014
Consolidation immunotherapy cycles	0.93 (0.89-0.96)	<0.001		

Abbreviations: BMI, body mass index; CI, confidence interval; COPD, chronic obstructive pulmonary disease; ECOG, Eastern Cooperative Oncology Group; GEJ, gastroesophageal junction; HR, hazard ratio; IC-CCRT, induction chemotherapy followed by concurrent chemoradiotherapy; IOC-CCRT, induction chemoimmunotherapy followed by concurrent chemoradiotherapy; PFS, progression-free survival; PGTV, planning gross tumor volume; TP, taxane plus platinum.
